# Role of Ca^2+^/calmodulin and PI3K/AKT signaling pathways and active ingredients of BaoTaiYin in treatment of recurrent miscarriage

**DOI:** 10.3389/fmolb.2025.1573294

**Published:** 2025-03-25

**Authors:** Li Ji, Anqi Deng, Huiying Chen, Shuangyan Guo, Pingyu Wang, Ruiyi Zhang, Wenyang Chen, Taotao Fan, Lijuan Jiang, Bing Shen

**Affiliations:** ^1^ The First Clinical Medical College, Nanjing University of Traditional Chinese Medicine, Nanjing, Jiangsu, China; ^2^ Gynecology, Lu’an Hospital of Traditional Chinese Medicine, Lu’an, Anhui, China; ^3^ School of Basic Medicine Sciences, Anhui Medical University, Hefei, China; ^4^ Central Laboratory, Fujian Medical University Union Hospital, Fuzhou, Fujian, China; ^5^ Department of Gynecology, The First Affiliated Hospital of Yunnan University of Traditional Chinese Medicine, Kunming, China; ^6^ Dr. Neher’s Biophysics Laboratory for Innovative Drug Discovery, State Key Laboratory of Quality Research in Chinese Medicine, Macau University of Science and Technology, Macau, China

**Keywords:** recurrent miscarriage, BaoTaiYin, trophoblast proliferation, Ca^2+^ signal, mass spectrometry, proliferation

## Abstract

**Introduction:**

BaoTaiyin (BTY) is a traditional Chinese medicine decoction. It has been used to treat recurrent miscarriage (RM). However, there are no comprehensive systematic studies to identify the chemical compositions of BTY and molecular mechanisms on RM. Finding the chemical components of BTY and clarifying the underlying processes in the treatment of RM were the goals of the study.

**Methods:**

We used ultra-high-performance liquid chromatography coupled with triple quadruple time-of-flight tandem mass spectrometry to analyze the chemical components of BTY, network analysis to predict the pharmacological effects of the identified active ingredients, and cell experiments to identify potential molecular mechanisms.

**Results:**

We found 12 active ingredients among 61 components identified in BTY. These identified activities were linked to regulatory effects on 127 key signaling pathways, targeting 107 proteins. Through network analysis, we determined that insulin-like growth factor 1 receptor, matrix metalloproteinases, PI3K, and STAT3 may be the core targets of BTY’s therapeutic effects on RM. We further explored this mechanism to find that aqueous extracts of BTY significantly enhanced IGFBP2 and CaMKK2 expression and trophoblast proliferation, whereas inhibitors of IGF1R/PI3K/AKT pathway or CaMKK2 blocked the effect of BTY on trophoblast proliferation. In addition, IGFBP2 siRNA suppressed BTY-induced CaMKK2 expression. Caffeic acid, as one of components of BTY, increased intracellular Ca^2+^ concentration and proliferation in trophoblast.

**Conclusion:**

Our research showed that BTY may have therapeutic benefits on RM through multiple targets and pathways, such as the IGF1R/PI3K/AKT and Ca^2+^/calmodulin signaling pathways.

## 1 Introduction

According to epidemiological data, 1%–3% of women of childbearing age experience recurrent miscarriage (RM), which is defined as the occurrence of two or more pregnancy losses (Quenby et al., 2021). The emotional and mental wellbeing of couples getting ready for pregnancy is obviously harmed by RM; therefore, it is important that they receive support and guidance from healthcare providers. According to previous studies, the causes of RM include thrombophilia, chromosomal abnormalities, coagulation disorder, metabolic and endocrine disorders, nutrition factors, anatomical malformations, and immunologic factors (McNamee et al., 2012; Quenby et al., 2021; van Wely, 2023). Although RM has a profound impact on individuals and society, there is still no good treatment for it. In the past, RM has largely relied on the use of aspirin and heparin to combat hypercoagulability, although only a few placebo-controlled trials have shown their benefit with respect to the live birth rate (Hamulyák et al., 2020). Therefore, it is imperative to develop novel treatments for RM.

In traditional Chinese medicine (TCM), RM is included in the categories of “miscarriage” and “repeated pregnancy and abortion.” The Chinese medicine BaotaiYin (BTY) is formed by adding SiJunZi Soup to the ShouTai Pill on the basis of information contained in *Integrating Chinese and Western Medicine* (Jiang et al., 2011; Zhang et al., 2022). Clinical studies have shown that for patients with antiphospholipid antibody–associated RM, BTY is a safe and effective prophylactic treatment (Pan and Dai, 2021). The ingredients of BTY include *Cuscuta australis* R.Br. [Convolvulaceae], *Atractylodes macrocephala* Koidz. [Asteraceae], *Codonopsis pilosula* (Franch.) Nannf. [Campanulaceae], *Eucommia ulmoides* Oliv. [Eucommiaceae], *Smilax glabra* Roxb. [Smilacaceae], *donkey-hide gelatin*, *Astragalus mongholicus* Bunge [Fabaceae], *Taxillus chinensis* (DC.) Danser [Loranthaceae], *Dipsacus asper* Wall. ex DC. [Caprifoliaceae] and *Glycyrrhiza glabra* L. [Fabaceae] among others. All plant names have been certified by MPNS (http://mpns.kew.org). However, certain components and pharmacodynamic properties of the constituents of BTY remain inadequately detected, identified, and characterized. This limitation significantly hinders our understanding of its pharmacology and pharmacodynamics, as well as its potential applications in medicine. As a result, a thorough and methodical examination of BTY’s chemical components is crucial. Additionally, further evaluation of its efficacy and elucidation of its mechanisms of action would be highly valuable.

Thus, the current study’s goals were to use network pharmacology to clarify BTY’s main components and related mechanisms of action in the treatment of RM and to investigate the active compounds in BTY using ultra-high-performance liquid chromatography coupled with quadrupole time-of-flight tandem mass spectrometry (UPLC-Q-TOF-MS/MS). We then evaluated one of the most convincing results from our studies using *in vitro* experiments.

## 2 Materials and methods

### 2.1 Experimental compound discovery

#### 2.1.1 Chemicals and materials

Jiangsu Kanion Pharmaceutical Co. Ltd., located in Lianyungang City, Jiangsu Province, China, provided the BTY herb. The herb was maintained at a temperature of 4 C till it was utilized in the research studies. Acetonitrile and methanol for mass spectrometry (MS) were procured from Merck (Darmstadt, Germany). Purified water was obtained from Watson’s Food & Beverages (Guangzhou, China), while formic acid for MS was sourced from Shanghai Aladdin Life Technology Co. (Shanghai, China). The ultra-high-performance liquid chromatograph was acquired from the Waters Corporation (Shanghai, China). The high-resolution mass spectrometer was purchased from Sciex (AB Sciex Triple TOF® 4600, California, United States). An electronic balance was obtained from Mettler Toledo International Trading Co. Ltd. (Shanghai, China). Additionally, an ultrasonic cleaner was procured from Kunshan Ultrasonic Instrument Co. Ltd. (Kunshan, China), and a high-speed centrifuge was purchased from Merck (Darmstadt, Germany).

#### 2.1.2 Sample preparation

BTY powder (0.5 g) was weighed, and placed into a 50 mL stoppered conical flask, along with 10 mL of 50% aqueous methanol, and sonicated (300 W, 40 kHz) at 37 °C for 30 min. The extract was removed, cooled, and shaken well. Afterwards, 2 mL of the extract was subjected to centrifugation at 12,000 rpm for 5 min. Next, the supernatant was filtered and analyzed qualitatively.

#### 2.1.3 UPLC-Q-TOF-MS/MS analysis

The chromatographic separation was conducted at a temperature of 30 °C utilizing Waters Acquity UPLC HSS T3 columns (2.1 × 100 mm, 1.8 µm). The mobile phases employed were acetonitrile (A) and an aqueous solution of 0.1% formic acid (B). The elution steps were as follows: (1) 0–3 min, 3% A; (2) 3–6 min, 3%–10% A; (3) 6–20 min, 10%–22% A; (4) 20–25 min, 22%–38% B; (5) 25–33 min, 38%–55% B; (6) 33–37 min, 55%–70% B; (7) 37–42 min, 70%–95% A; (8) 42–45 min, 95% A; (9) 45–45.1 min, 95%–3% A; and (10) 45.1–48 min 3% A. The data acquisition software utilized was Analyst (version 1.7.1). In the data analysis, the processing software employed was PeakView (version 1.2).

The mass spectrometry (MS) detection was performed in both ESI-Negative and Positive ion modes, with the MS parameters detailed in Table 1. The identification process prioritized matching the MS data against the Natural Products High Resolution MS/MS Spectral Library (1.0) database. In our data, compounds were initially obtained dependent on the peak score information, followed by further confirmation utilizing both first-order and second-order information for each peak.

**TABLE 1:** Mass spectrometry (MS) parameters used in the study.

MS parameter	Parameter value	MS/MS parameter	Parameter value
TOF mass range	50–1700	MS/MS mass range	50–1,250
Ion Source Gas 1 (psi)	50	Declustering Potential (V)	100
Ion Source Gas 2 (psi)	50	Collision Energy (eV)	±40
Curtain Gas (psi)	35	Collision Energy Spread (eV)	20
Ion Spray Voltage Floating (V)	−4500/5000	Ion Release Delay (ms)	30
Ion Source Temperature (°C)	500	Ion Release Width (ms)	15
Declustering Potential (V)	100		
Collision Energy (eV)	10		

Note: The instrument used was AB, Sciex’s Triple TOF, 4600 LC-MS. TOF, represents time-of-flight.

The Natural Products High Resolution MS/MS Spectral Library (1.0) database (https://sciex.com/kr/products/spectral-library/hrms-natural-products) encompasses multistage mass spectra of our standards as well as those from other sources, incorporating various acquisition modes, added ions, and collision energies. For compounds not included in this database, identification was achieved through analysis of the multilevel mass spectra obtained from samples, supplemented by relevant literature and comparisons with entries in the aforementioned database.

### 2.2 Predicting the molecular targets of compounds in BTY

We screened all compounds identified in BTY that exhibited an “oral bioavailability (OB) ≥30%,” as reported in TCMSP database (https://old.tcmsp-e.com/tcmsp.php), or were classified as “able to be absorbed into the blood” according to SwissADME (http://www.swissadme.ch/). The active ingredients were subsequently obtained. The canonical SMILES of these compounds were searched from the database of PubChem (https://pubchem.ncbi.nlm.nih.gov) or NovoPro (https://www.novopro.cn/tools/mol2smiles.html). We then uploaded the canonical SMILES of these compounds to both the databases of SwissTargetPrediction (www.swisstargetprediction.ch, updated 2019) and STITCH (http://stitch.embl.de/, version 5.0) to acquire UniProt IDs to predict molecular targets relevant to *Homo sapiens*.

### 2.3 Obtaining targets related to RM

Targets were obtained in the GeneCards database (https://www.genecards.org/) using the keywords “recurrent spontaneous abortion,” “recurrent abortion,” and “miscarriage.” Similarly, “recurrent spontaneous abortion” was searched in DisGeNET (https://disgenet.com/) and “recurrent abortion” and “miscarriage” were the keywords searched in the OMIM database (https://www.omim.org/) to obtain targets. We combined the acquired disease targets and removed the duplicates to finally obtain the targets related to RM.

### 2.4 Identifying potential targets for BTY against RM by bioinformatics

To identify all potential target genes common to BTY and RM, the intersection for drug and disease target genes was analyzed using the Venny (version 2.1.0) online tool (https://bioinfogp.cnb.csic.es/tools/venny/), resulting in a Venn diagram illustrating the overlapping targets. Subsequently, a network of protein-protein interaction (PPI) for these targets was generated utilizing the STRING platform (https://cn.string-db.org/) alongside Cytoscape (version 3.10.0) software. The key targets were selected dependent on their degree values. Enrichment analysis of the drug/disease shared core targets was performed using the DAVID database (https://david.ncifcrf.gov/, *P* ≤ 0.05 as the screening criterion). Gene Ontology (GO) enrichment analysis histograms and Kyoto Encyclopedia of Genes and Genomes (KEGG) enrichment maps were plotted using the microbiology platform on Wei Sheng Xin (https://bioinformatics.com.cn/). To fully clarify the molecular mechanisms underlying the treatment of RM with BTY, we used Cytoscape (version 3.10.0) to construct active ingredient–shared pathway targets. In the network, the connect nodes indicate compounds, some diseases, molecular targets, or some signaling pathways, while edges denote the interactions among them.

### 2.5 Cell culture and proliferation experiments

HTR-8/SVneo cells as a human chorionic trophoblast cell line were obtained from the American Type Culture Collection (ATCC; Manassas, VA, United States). The cells were cultured in RPMI-1640 medium (BasalMedia, Shanghai, China) supplemented with 10% fetal bovine serum (#A5669401, Thermo Fisher Scientific, Waltham, United States) and 1% penicillin-streptomycin. Cultivation was performed at 37 °C under saturated humidity and a CO_2_ concentration of 5%. Cell proliferation was evaluated using a CCK8 kit (#C0037, Beyotime, Shanghai, China). Cells were seeded into a 96-well plate, and the absorbance was measured at 450 nm wavelength. The cells received treatment with aqueous extracts of BTY alone at various concentrations or in combination with either 0.2 μM picropodophyllin (#407247, Merk SA, Darmstadt, Germany), 0.1 μM ZSTK474 (#SML3194, Merk SA, Darmstadt, Germany), or 0.2 μM afuresertib (#A413769, Aladdin Biochemical Technology Co., Ltd., Shanghai, China).

### 2.6 Reverse transcription-quantitative PCR (RT-qPCR)

Using a cDNA Reverse Transcription Toolkit (#11141ES60, Yeasen Biotechnology Co., Ltd., China), we added entire RNA that had been isolated from several cell types to create cDNA. A TB Green Premix Ex TaqTM II kit (#RR820A TaKaRa Bio; Japan) and Roche LightCycler 480 II PCR equipment (Roche, Switzerland) were utilized for the qPCR process (5 min at 95 C, followed by 40 cycles of 15 s at 95 C, 30 s at 61 C). The primers utilized in this study were the same as those used in a previous one (Ji et al., 2024). The primers for the genes are listed in Table 2. The 2^−ΔΔCT^ procedure was utilized to quantify the mRNA level in relation to β-actin/18S rRNA.

**TABLE 2:** Primer sequences for the RT-qPCR analysis.

Gene	Forward primers (5′-3′)	Reverse primers (5′-3′)
ACTB	TCCACGAAACTACCTTCAACTCCAT	ATACTCCTGCTTGCTGATCCACATC
PCNA	ACACTAAGGGCCGAAGATAACG	CAGCATCTCCAATATGGCTGA
IGFBP2	GACAATGGCGATGACCACTCA	CAGCTCCTTCATACCCGACTT

### 2.7 Western blotting

Cells were lysed in RIPA buffer. The total proteins were separated on 10% electrophoretic SDS gels and transferred onto polyvinylidene difluoride membranes. The non-specific binding was blocked with a PBST buffer. Next, the membranes containing proteins were incubated with Ca^2+^/calmodulin-dependent protein kinase kinase 2 (CaMKK2) antibodies (#aa43-71, LifeSpan BioSciences, Inc. Seattle, WA. United States) at 4 °C overnight. In next day, the blot membranes were treated with horseradish peroxidase-conjugated secondary antibody (1:5000). Eventually, the immunosignals were developed by using an ECL system. GAPDH protein was used as a loading protein control.

### 2.8 Intracellular Ca^2+^ concentration measurement

We used a previous protocol to measure intracellular Ca^2+^ concentration ([Ca^2+^]_i_) (Dai et al., 2021). Briefly, HTR-8/SVneo cells were seeded on some cover slips and cultured in 12-well plates before experiments. To load Ca^2+^ fluorescent dye, we incubated the cells for 20 min at 37 C with 2 mM Fluo-8/AM (#1345980-40-6, Abcam company, Shanghai, China) and 0.02% pluronic F-127 (#P3000MP, Thermo Fisher Scientific, Waltham, United States). The increase of [Ca^2+^]_i_ were measured in a physiological saline solution (140 mM NaCl, 5 mM KCl, 2 mM CaCl_2_, 1 mM MgCl_2_, 10 mM glucose, and 5 mM HEPES, pH 7.3 to 7.4 adjusted with NaOH) through a fluorescence microscope (Nikon T200, Tokyo, Japan). The fluorescent baseline signal before addition of agonist was F0, and the change of [Ca^2+^]_i_ is expressed as the ratio of fluorescent intensity relative to the baseline signal (F1/F0).

### 2.9 Statistical analysis

The GraphPad Prism (version 7.0) statistical software was used to analyze the data, and one-way analysis of variance was conducted. In our data, the values are expressed as mean ± SE. Values of *P* < 0.05 were considered statistically significant.

## 3 Results

### 3.1 Identification of chemical constituents in BTY

**FIGURE 1:**
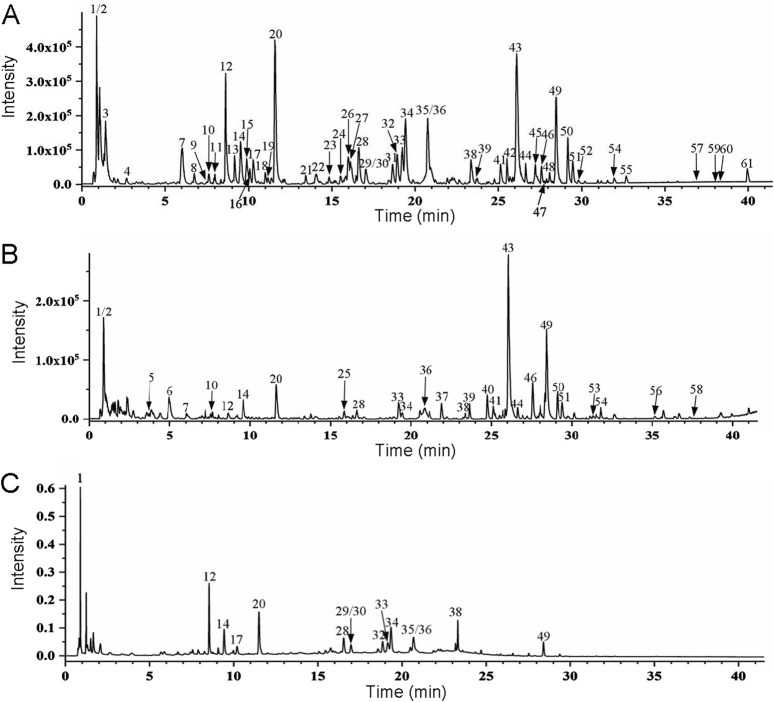
UPLC high-resolution MS base peak chromatogram showing BaoTaiYin ion intensities in negative ion mode **(A)** and positive ion mode **(B)**. **(C)**. Ultraviolet chromatogram of BaoTaiYin using UPLC-ultraviolet at a wavelength of 254 nm.

UPLC-Q-TOF-MS/MS was used to collect the active chemical components in BTY, and the base peak and frontal chromatograms using positive (Figure 1A) or negative (Figure 1B) ion mode, and UPLC-ultraviolet (Figure 1C) were obtained. From the MS data, we identified a total of 61 compounds, including saccharides, organic acids, phenolic acids, amino acids, alkaloids, cycloalkenyl ether terpene glycosides, lignans, flavonoid glycosides, acetylenic glycosides, triterpene saponins, fatty acids, glycosides, sesquiterpenoids, and triterpenoids. The detailed results are given in Supplementary Table S1.

### 3.2 Analysis of network pharmacology

#### 3.2.1 Identification of BTY-associated targets and analysis of disease-target genes

**FIGURE 2:**
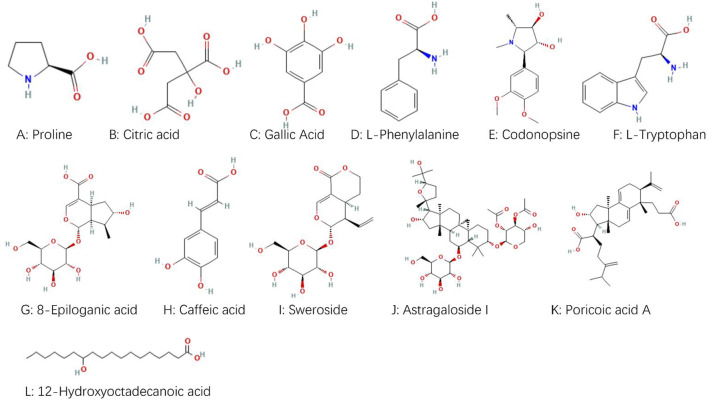
Chemical structures for 12 active ingredients in BaoTaiYin.

The chemical components identified in BTY were screened using the criterion “OB ≥ 30%” or “able to be absorbed into the blood,” and 12 active ingredients were obtained: proline, citric acid, gallic acid, L-phenylalanine, codonopsis alkaloids, L-tryptophan, 8-epiloganic acid, caffeic acid, sweroside, astragaloside I, porinic acid A, and 12-hydroxystearic acid. Their chemical structures are shown in Figure 2. The target genes of each chemical component were extracted in the TCMSP and Swiss Target Prediction databases. After merging the data and eliminating duplicate entries, 289 target genes were found. Using “miscarriage” as a keyword obtained 2,148 targets. Searching with “recurrent spontaneous abortion” as a keyword in DisGeNET returned 429 targets, and searching with “recurrent abortion” and “miscarriage” as keywords in the OMIM database returned 102 targets. After combining the captured disease targets and removing the duplicates, we finally obtained 2,379 targets for RM.

#### 3.2.2 PPI network analysis

**FIGURE 3:**
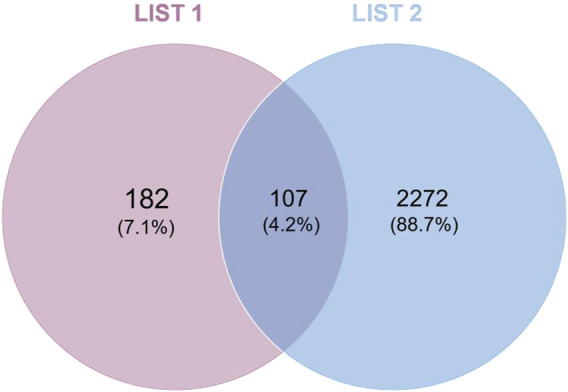
Wayne diagram showing the intersection of the disease targets and drug targets. The pink section is the drug target, the blue section is the disease target, and their intersection contains the targets common to both.

**FIGURE 4:**
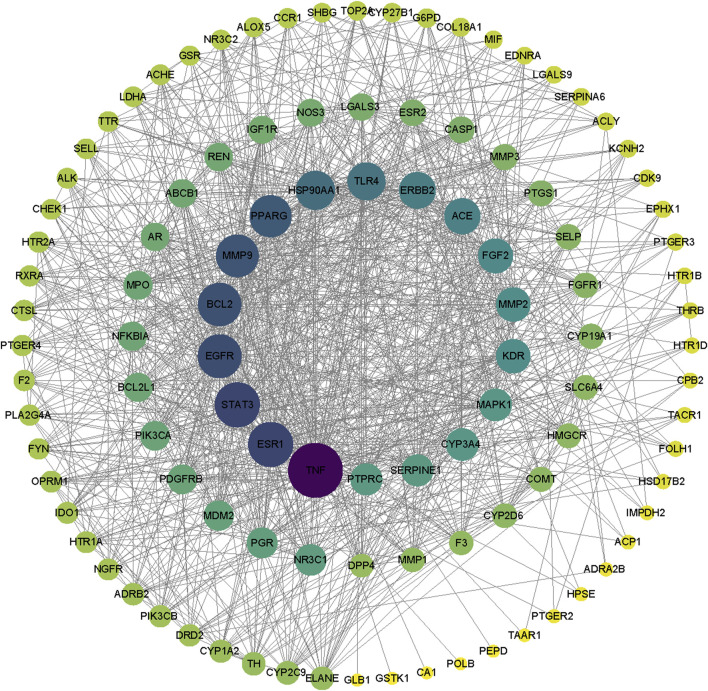
Protein-protein interaction network. Nodes with darker colors and larger sizes are considered higher degree targets.

Mapping the drug targets and disease targets using a Wayne diagram (Figure 3) identified 107 shared targets. A PPI network of targets from the intersection of the Wayne diagram was constructed with the STRING platform and Cytoscape 3.7.0 software. The results showed 108 nodes and 1,067 edges, with an average number of neighbors of 20.132 (Figure 4). The targets were ranked and analyzed based on their degree value. We identified the following 24 key targets from the PPI network with thresholds of closeness ≥0.005, betweenness ≥106.925, and degree ≥20.132: ABCB1, NR3C1, LGALS3, SLC6A4, KDR, STAT3, ERBB2, EGFR, HSP90AA1, MMP9, BCL2, TNF, ESR1, CYP2D6, CYP3A4, ACE, MAPK1, SERPINE1, FGF2, PPARG, TLR4, PTGS1, PGR, and AR. These 24 proteins may be potential key targets for the treatment of RM with BTY.

#### 3.2.3 GO and KEGG analysis

**FIGURE 5:**
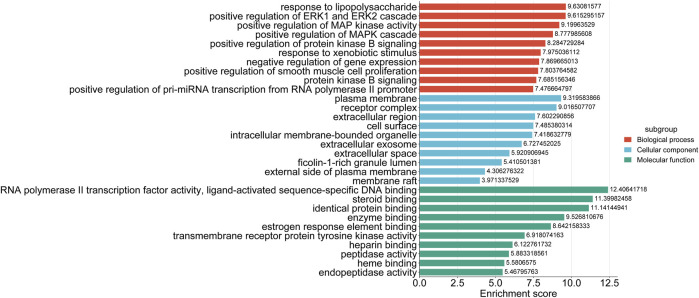
Top 10 enriched gene ontology terms for the biological processes, cellular components, and molecular functions of the potential targets.

**FIGURE 6:**
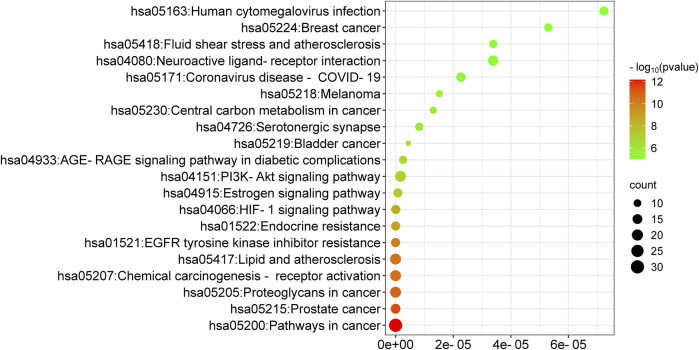
Kyoto Encyclopedia of Genes and Genomes enrichment analysis of top 20 pathways. FDR represents false discovery rate.

GO functional annotation and KEGG pathway analysis were performed by assessing the targets found at the intersection of the targets for drug and disease on the DAVID platform. GO functional annotation utilizes biological processes, molecular functions, and cellular components to annotate and classify genes. This analysis yielded 412 biological process annotations, 50 cellular component annotations, and 96 molecular function annotations. The top 10 items in each group were used for mapping (Figure 5). It was found that the biological processes were mainly involved in the positive regulation of ERK1 and the ERK2 cascade response, positive regulation of MAP kinase activity, positive regulation of smooth muscle cell proliferation, positive regulation of cell proliferation, and multicellular biogenesis. The cellular components mainly comprised the cell membrane, cellular exosome, azurophilic granule lumen, lysosome lumen, macromolecular complex, and synapse. The molecular functions were primarily associated with the RNA polymerase II transcription factor activity, estrogen response element binding, heparin binding, heme binding, steroid hormone receptor activity, and oxidoreductase activity. A total of 127 KEGG pathways were enriched, and the top 20 were retained in the order of their *P* value. These pathways included pathways in cancer (hsa05200), the HIF-1 signaling pathway (hsa04066), PD-L1 expression and PD-1 checkpoint pathway in cancer (hsa05235), apoptosis (hsa04210), and other signaling pathways (Figure 6). In the GO and KEGG results, we identified a series of molecular functions and pathways related to cell proliferation, immune response, oxidative stress, blood coagulation, and others that had a high degree of similarity. In the GO analysis results, many molecular functions were related to cell proliferation, including matrix metalloproteinases (MMPs), signal transduction and transcription activation protein-3 (STAT3), epidermal growth factor receptor (EGFR), fibroblast growth factor 2 (FGF2), and kinase insertion structural domain receptor (KDR). Similarly, the findings of the KEGG analysis showed a number of pathways, such as the cAMP, Ca2+, and PI3K-AKT signaling pathways, that were linked to cell proliferation. These results suggested that encouraging cell proliferation is the most likely way that BTY may cure RM.

#### 3.2.4 Examination of the active ingredient–shared target–pathway network

**FIGURE 7:**
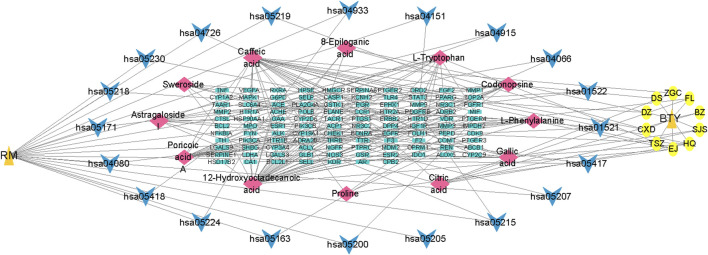
Active ingredient–shared target–pathway network analysis. Orange triangles represent the disease (recurrent miscarriage, RM) and the drug (BaoTaiYin, BTY), yellow circles represent the herbs present in BTY, blue arrows represent pathways, red diamonds represent active ingredients, and green rectangles represent targets.

Using the top 20 signaling pathways from the KEGG pathway enrichment analysis, we constructed the linkages between the active ingredients, pathway targets, and signaling pathways, and visualized them with Cytoscape 3.7.2 software. As shown in Figure 7, the network had 151 nodes and 208 edges, including 12 active compounds, 107 targets, and 20 pathways. The active ingredient–shared target–pathway network revealed the synergistic therapeutic effects between multiple active ingredients and targets at the intersection of disease (RM) and drug (BTY), as well as the interactions between these targets and pathways, reflecting the holistic nature underlying the efficacy of TCM.

### 3.3 Role of the IGF1R-PI3K-AKT signaling pathway in BTY-induced trophoblast cell proliferation

**FIGURE 8:**
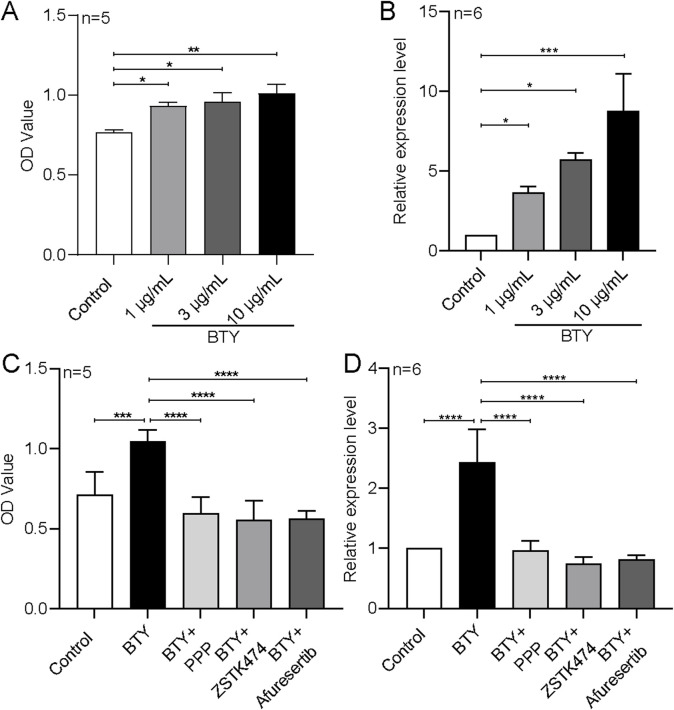
Effect of BaoTaiYin (BTY) on the expression of insulin-like growth factor-binding protein 2 (IGFBP2) and the impact of inhibitors targeting the insulin-like growth factor 1 receptor (IGF1R)-PI3K-AKT signaling pathway on BTY-induced proliferation of HTR-8/SVneo cells. Summary data illustrating cell viability **(A and C)**, as assessed by the CCK8 assay, along with relative mRNA expression levels **(B and D)** for IGFBP2 **(B)** and proliferating cell nuclear antigen gene (PCNA, **(D)** in HTR-8/SVneo cells treated with solvent control, 10 μg/mL BTY, BTY combined with picropodophyllin (PPP, 0.2 μM), BTY combined with ZSTK474 (0.1 μM; a PI3K inhibitor), or BTY combined with afuresertib (0.2 μM; an Akt inhibitor) for a duration of 48 h. OD represents relative optical density. The mRNA levels were normalized to that of 18S rRNA. Data represent the mean ± SE. *P < 0.05, **P < 0.01, ***P < 0.001, ****P < 0.0001 for the indicated comparison.

According to our team’s earlier research, patients with RM have much lower levels of insulin-like growth factor-binding protein 2 (IGFBP2) in their plasma. IGFBP2 acts on the insulin-like growth factor 1 receptor (IGF1R)-PI3K-AKT signaling pathway to promote trophoblast cell proliferation (Ji et al., 2024). The current study’s KEGG analysis showed that BTY has the ability to control the PI3K-AKT signaling pathway. Therefore, we tested the functional effects of BTY on cell proliferation, IGFBP2 expression, and the participation of IGFBP2 downstream of the IGF1R-PI3K-AKT signaling cascade using a human trophoblast cell line (HTR-8/SVneo). According to our findings, BTY aqueous extracts markedly increased HTR-8/SVneo cell proliferation and IGFBP2 expression (Figures 8A,B). By inhibiting the IGF1R-PI3K-AKT signaling pathway one inhibitor at a time with the IGF1R inhibitor picropodophyllin (PPP, 0.2 μM), the PI3K inhibitor ZSTK474 (0.1 μM), and the AKT inhibitor afuresertib (0.2 μM), we discovered that all three inhibitors significantly decreased the BTY-induced increase in proliferation and inhibited the BTY-induced increase in mRNA expression of the proliferation marker PCNA in HTR-8/SVneo cells (Figures 8C,D).

### 3.4 Role of Ca^2+^/calmodulin signaling pathway in BTY-induced trophoblast cell proliferation

**FIGURE 9:**
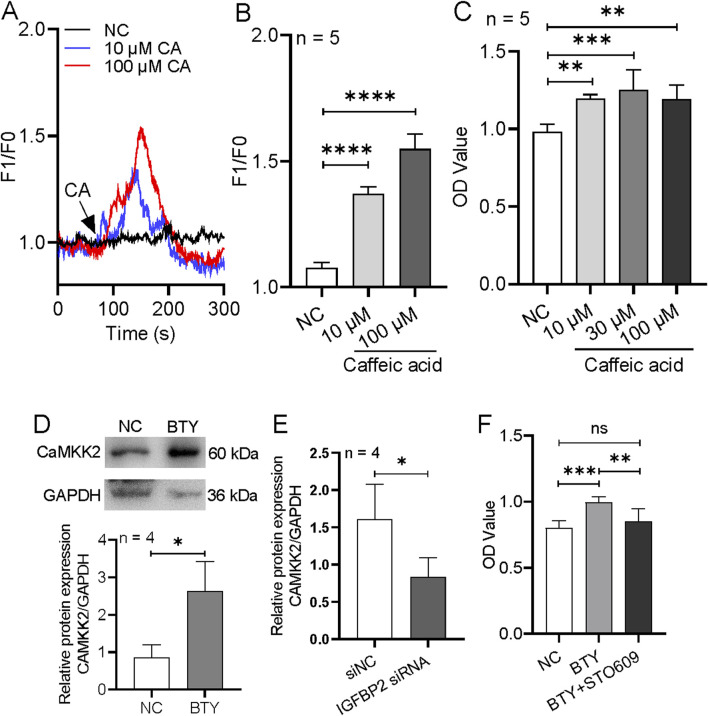
Effect of caffeic acid on proliferation and intracellular Ca^2+^ rise and role of CaMKK2 in BaoTaiYin (BTY)-induced proliferation in HTR-8/SVneo cells. **(A, B)**, Representative traces and summary data showing 10 and 100 µM caffeic acid (CA)-induced intracellular Ca^2+^ concentration increase. **(C)**, Summary data showing the proliferation of HTR-8/SVneo cells treated with solvent control (NC) and caffeic acid with 10, 30 and 100 µM concentration. **(D-E)**, Representative images and summary data showing the expression levels of CaMKK2 in cells treated with or without BTY (10 μg/mL) **(D)**, or transfected with control siRNA (siNC) or IGFBP2 siRNA and treated with BTY (10 μg/mL) **(E)**. **(F)**, Summary data showing the proliferation of HTR-8/SVneo cells treated with solvent control (NC), BTY (10 μg/mL) or BTY (10 μg/mL) + STO609 (5 μg/mL). Data represent the mean ± SE. *P < 0.05, **P < 0.01, ***P < 0.001, ****P < 0.0001 for the indicated comparison.

In components of BTY, caffeic acid has effect to induce [Ca^2+^]_i_ rise in cells (Chang et al., 2013). Therefore, we also tested the effect of caffeic acid on the regulation of [Ca^2+^]_i_ and cell proliferation in HTR-8/SVneo cells. Our results indicated that 10 and 100 μM caffeic acid strongly increased [Ca^2+^]_i_, and furthermore 10–100 μM caffeic acid treatment significantly enhanced cell proliferation (Figures 9A–C).

Calmodulin is a well-known downstream of Ca^2+^ signaling. Xi et al. reported that IGFBP2 also can stimulate CaMKK2 activity (Lu et al., 2022). Therefore, we examined CaMKK2 expression and effect of CaMKK2 on the proliferation in HTR-8/SVneo cells. Our results indicated that BTY treatment significantly enhanced CaMKK2 expression, but if we used IGFBP2 siRNA to suppress IGFBP2 expression, BTY-induced CaMKK2 expression increment was removed (Figures 9D,E). In addition, the treatment of CaMKK2 inhibitor (STO609) significantly reduced BTY-induced cell proliferation (Figure 9F). Therefore, these data indicated that BTY may enhance cellular [Ca^2+^]_i_ to induce cell proliferation by Ca^2+^/calmodulin signaling pathway.

## 4 Discussion

There is currently no universally accepted international definition of RM. However, according to some current recommendations, RM is defined as two or more consecutive pregnancy losses occurrence—including biochemical pregnancies (Dimitriadis et al., 2020). The risk of re-pregnancy miscarriage in individuals with RM, a frequent pregnancy complication, can range from 40% to 80% (Green and O’Donoghue, 2019; Deng et al., 2022). According to surveys, the number of previous miscarriages and pregnant woman age are identified as the primary risk factors for RM. However, other factors such as embolism, alcohol consumption, stress, night-shifts, environmental pollution, and exposure to toxic chemical substances are also currently regarded as plausible risk factors (Quenby et al., 2021). However, the multidimensional nature of the etiology of RM along with a lack of specific clinical manifestations prior to RM have hindered progress in the treatment of RM, and empirical and combined multiple treatments remain the standard of care (Deng et al., 2022). Among the various symptom types in traditional Chinese medicine, RM is classified under several syndromes, including kidney deficiency with blood stasis, spleen and kidney deficiency, isolated kidney deficiency, qi-blood weakness, yin deficiency, and blood heat syndrome. (Sun et al., 2024). BTY is effective in strengthening the spleen and toning the kidney, clearing away heat and tranquilizing the fetus, resolving blood stasis, and nourishing blood, and it is commonly used in the treatment of preterm labor, habitual abortion, and preterm labor (Shen, 2017). The findings of the present study suggest multiple mechanisms through which BTY may be useful in the treatment of RM as well provide some guidance for the study of its clinical dosing characteristics.

UPLC-Q-TOF-MS/MS is a highly sensitive detection method that has been extensively employed as an effective tool for the rapid screening and systematic identification of chemical components in TCM (Tian et al., 2025). Using UPLC/Q-TOF-MS/MS, we identified 61 compounds in BTY extracts, including 10 flavonoid glycosides, 15 triterpenoids and their glycosides, 12 phenolic acids, five alkaloids, six cyclic enol ether terpene glycosides, three sesquiterpenoids, two fatty acid compounds, three amino acids, one organic acid, one alkynyl glycoside, lignans, one sugar and a glucoside. Network pharmacology represents a significant research methodology within systems biology, characterized by its holistic and integrative approach. This network pharmacology framework analyzes the interactions of compounds with various targets, cells, and organs at molecular and genetic levels through network visualization techniques. Such analysis elucidates the intricate biological networks connecting compounds, genes, and targets (Niu et al., 2021; Li et al., 2023). By utilizing this network pharmacology approach and constructing “active ingredient–shared target–pathway” networks based on the structural properties and therapeutic effects of compounds, the activities and mechanisms of components in BTY can be effectively predicted. Based on these identified compounds, we obtained 107 potential targets using the STING database, including the following. A multipurpose biological molecule, tumor necrosis factor (TNF) is essential for inflammation, apoptosis, and immune system development (Fischer et al., 2020). MMPs are crucial in processes including cell migration, apoptosis, and angiogenesis. Of them, MMP2 and MMP9 are thought to be strongly linked to the Chinese Han population’s risk of RM (Li et al., 2018). The cell cycle is significantly impacted by STAT3, and apoptosis is induced when the STAT3 pathway is inhibited (Zhao et al., 2023). Through the mediation of estrogen and progesterone, estrogen receptor-1 (ESR1) is essential for the preservation of pregnancy (Huang et al., 2024). In connection with placenta formation, EGFR is linked to the proliferation and differentiation of placental trophoblast cells (Karachrysafi et al., 2023). Toll-like receptor 4 (TLR4) is a significant class of protein molecules that plays a crucial role in non-specific immunity (natural immunity) and is believed to be instrumental during early pregnancy (Kolben et al., 2019). The renin-angiotensin-aldosterone system depends on the zinc-dependent dipeptidyl carboxypeptidase known as angiotensin-converting enzyme (ACE). Additionally, ACE is involved in immune regulation and has been demonstrated to have a causal relationship with prothrombogenicity (Miljanović et al., 2023). FGF2 has the ability to promote cellular activity and cellular mitosis (Nawrocka et al., 2020). KDR is an important gene in the control of angiogenesis (Honarvar et al., 2016). Upregulation of suppressor of human fibrinogen activator-1 (SERPINE1) leads to a decrease in fibrinolytic activity, which in turn contributes to thrombophilia (Khosravi et al., 2014). The progesterone receptor (PGR) mediates progesterone and plays a key role in the maintenance of pregnancy (Huang et al., 2024). P-glycoprotein (ABCB1) is an efflux pump that harnesses the energy derived from ATP to transport a wide array of structurally diverse compounds out of the cell, and has been associated with idiopathic RM due to oxidative stress (Khadzhieva et al., 2014). Galectin-3 (LGALS3) is associated with oxidative stress in cells associated with lysosomal rupture during cellular autophagy (Yao et al., 2021). Cyclooxygenase 1 (PTGS1) catalyzes the conversion of arachidonic acid to prostaglandins, which acts a pivotal part in both physiological and pathological processes, encompassing inflammation, pain, and thrombosis (Li et al., 2021). CYP2D6 is an important drug metabolism–associated enzyme, catalyzing the oxidation of testosterone to androstenedione, which increases during pregnancy (Yousefian et al., 2022). These findings demonstrate that BTY have the potential to treat RM by modulating the aforementioned targets. This modulation could subsequently lead to the regulation of oxidative stress, inhibition of embolism, promotion of cell mitosis, and adjustment of sex hormones.

In addition to the identified potential targets, our constructed PPI network, along with GO and KEGG analyses, revealed that the PI3K-AKT signaling pathway, Ca^2+^ signaling pathway, cAMP pathway, apoptosis, and other related pathways are common targets for both BTY and RM. Several studies have shown that the signaling pathway of PI3K-AKT regulates a number of cellular functions, including angiogenesis, cell growth, and apoptosis; inhibition of this pathway has been shown to hinder the growth and invasion of human trophoblast cells, which in turn affects human placentation and the maintenance of early pregnancy (Li et al., 2025). Moreover, the signaling pathway of PI3K-AKT has been linked to an increase in the secretion of vascular endothelial growth factor within trophoblast cells and is essential for controlling chorionic trophoblast function (Chang et al., 2024). In the meantime, the Ca^2+^ signaling pathway is important for driving fertilization and initiating developmental processes (Walker, 2025). Related research has discovered variations in intracellular free Ca^2+^ concentrations in erythrocytes, platelets, and immune cells throughout pregnancy (Adamova et al., 2009; Albaghdadi et al., 2024). Additionally, Ca^2+^-dependent matrix metalloproteinases may also encourage the breakdown of extracellular matrix and vascular remodeling during pregnancy (Qu and Khalil, 2020). Finally, prostaglandin E2 receptor four affects trophoblast function by triggering the cAMP-PKA-pCREB signaling cascade at the maternal-fetal interface. This activation causes luteinizing hormone, interleukin-6, and β-human chorionic gonadotropin levels to drop (Peng et al., 2021). Another crucial aspect of RM is apoptosis, a strictly regulated form of cellular suicide (Li et al., 2020). BTY may have a therapeutic effect on RM by altering these pathways. BTY may modulate these pathways, resulting in a therapeutic effect on RM. In addition, a previous study by our team indicates that IGFBP2 expression is downregulated in patients with RM (Ji et al., 2024). As evidenced by transcriptional sequencing studies and trophoblast proliferation assays, IGFBP2 may affect pregnancy outcomes via adjusting trophoblast proliferation through the signaling pathway of PI3K-AKT. In order to learn more about IGFBP2 signaling and how BTY regulates it, we conducted further tests. We discovered that aqueous extracts of BTY significantly boosted both IGFBP2 expression and trophoblast proliferation and that inhibitors that the impact of BTY on trophoblast proliferation was eliminated by inhibitors that disrupted several elements of the signaling pathway of IGF1R-PI3K-AKT. Therefore, we followed up with experiments to further explore IGFBP2 signaling and its regulation by BTY. These results imply that BTY can help cure RM by influencing the signaling pathway of IGF1R-PI3K-AKT through IGFBP2, which would encourage trophoblast proliferation (Supplementary Figure S1). According to previous studies, caffeic acid can regulate Ca^2+^ concentration in cells (Chang et al., 2013). Furthermore, IGFBP2 can increase the activity of CaMKK2, which is one isoform of calmodulin as a downstream of Ca^2+^ signal (Lu et al., 2022). We found that BTY aqueous extracts significantly increased both IGFBP2 expression and trophoblast proliferation, and that inhibitors that targeted distinct components of the signaling pathway of IGF1R-PI3K-AKT reversed the effect of BTY on trophoblast proliferation. We found that BTY can increase cellular Ca^2+^ concentration through the Ca^2+^/calmodulin signaling pathway to induce trophoblast proliferation, but this induction is inhibited by suppressing IGFBP2 expression. Additionally, BTY significantly increased the expression of a Ca^2+^/calmodulin-activated kinases (CaMKK2), while suppression of IGFBP2 by specific siRNA abolished BTY-increased CaMKK2 expression similar to a previous study (Lu et al., 2022). CaMKK2 inhibitor significantly decreased BTY-induced trophoblast proliferation. Caffeic acid, as one component of BTY, strongly increased [Ca^2+^]_i_ and significantly promoted trophoblast proliferation. Therefore, BTY may regulate trophoblast proliferation by modulating intracellular Ca^2+^ signaling as well as the Ca^2+^/calmodulin pathway (Supplementary Figure S1). Additionally, in the components of BTY, reports from other groups showed that L-proline stimulates Ca^2+^ entry via activation of excitatory amino acid receptor (Henzi et al., 1992); gallic acid inhibits mesaconitine-induced TRPV1 activation (Han et al., 2022); L-phenylalanine activates G_q_-coupled receptor GPR142 to increase [Ca^2+^]_i_ (Osuga et al., 2022); astragalosides prevents isoproterenol-induced increase in [Ca^2+^]_i_ and sarcoplasmic reticulum Ca^2+^ load (Meng et al., 2005). These findings indicate that BTY may have complex effect on [Ca^2+^]_i_ regulation, and maybe alleviates RM through multiple pathways and mechanisms.

But this study has some limitations. First, some components of BTY may have been overlooked during the component identification process. Second, the herbal chemistry database is continually being updated and refined. Furthermore, for a more thorough knowledge of BTY’s methods of action *in vitro and in vivo*, greater investigation and confirmation of the main biological components and the targets they are linked to are necessary.

## 5 Conclusion

In this study, we employed UPLC/Q-TOF-MS/MS analysis and network pharmacology to investigate the mechanisms underlying the therapeutic effects of BTY on RM. Our findings indicate that 12 out of the 61 identified compounds in BTY may possess biological activity, with their effects potentially linked to 127 significant signaling pathways through interaction with 107 targets, including modulation via the IGF1R-PI3K-AKT and Ca^2+^/calmodulin signaling pathways. Further experimental data uncovered the effect and mechanism of BTY and its important component of caffeic acid on trophoblast proliferation. These results underscore the multifaceted components, targets, and pathways typically associated with traditional Chinese medicine. This research provides valuable insights into the molecular mechanisms related to BTY’s efficacy in treating RM.

## Statement

The name of Chinese herbal medicine has been checked in World Flora Online (www.worldfloraonline.org) or MPNS (http://mpns.kew.org).

## Data Availability

The original contributions presented in the study are included in the article/Supplementary Material, further inquiries can be directed to the corresponding author.

## References

[B1] Adamova Z. Ozkan S. Khalil R. A. (2009). Vascular and cellular calcium in normal and hypertensive pregnancy. Curr. Clin. Pharmacol. 4, 172–190. 10.2174/157488409789375320 19500073 PMC2852626

[B2] Albaghdadi A. J. H. Xu W. Kan F. W. K. (2024). An immune-independent mode of action of tacrolimus in promoting human extravillous trophoblast migration involves intracellular calcium release and F-actin cytoskeletal reorganization. Int. J. Mol. Sci. 25, 12090. 10.3390/ijms252212090 39596157 PMC11593602

[B3] Chang H.-T. Chen I.-L. Chou C.-T. Liang W.-Z. Kuo D.-H. Shieh P. (2013). Effect of caffeic acid on Ca(2+) homeostasis and apoptosis in SCM1 human gastric cancer cells. Arch. Toxicol. 87, 2141–2150. 10.1007/s00204-013-1075-8 23685796

[B4] Chang R. Su Y. Kong H. Wang F. Xing Y. Jiang L. (2024). Upregulation of SEMP1 contributes to improving the biological functions of trophoblast via the PI3K/AKT pathway in preeclampsia. Mol. Biotechnol. 66, 531–543. 10.1007/s12033-023-00774-3 37277581

[B5] Dai F. Guo J. Wang Y. Jiang T. Chen H. Hu Y. (2021). Enhanced store-operated Ca2+ signal of small intestinal smooth muscle cells accelerates small bowel transit speed in type 1 diabetic mouse. Front. Physiol. 12, 691867. 10.3389/fphys.2021.691867 34744757 PMC8564290

[B6] Deng T. Liao X. Zhu S. (2022). Recent advances in treatment of recurrent spontaneous abortion. Obstet. Gynecol. Surv. 77, 355–366. 10.1097/OGX.0000000000001033 35672876 PMC9169761

[B7] Dimitriadis E. Menkhorst E. Saito S. Kutteh W. H. Brosens J. J. (2020). Recurrent pregnancy loss. Nat. Rev. Dis. Prim. 6, 98. 10.1038/s41572-020-00228-z 33303732

[B8] Fischer R. Kontermann R. E. Pfizenmaier K. (2020). Selective targeting of TNF receptors as a novel therapeutic approach. Front. Cell Dev. Biol. 8, 401. 10.3389/fcell.2020.00401 32528961 PMC7264106

[B9] Green D. M. O’Donoghue K. (2019). A review of reproductive outcomes of women with two consecutive miscarriages and no living child. J. Obstet. Gynaecol. 39, 816–821. 10.1080/01443615.2019.1576600 31006300

[B10] Hamulyák E. N. Scheres L. J. Marijnen M. C. Goddijn M. Middeldorp S. (2020). Aspirin or heparin or both for improving pregnancy outcomes in women with persistent antiphospholipid antibodies and recurrent pregnancy loss. Cochrane Database Syst. Rev. 5, CD012852. 10.1002/14651858.CD012852.pub2 32358837 PMC7195627

[B11] Han S. Bao L. Li W. Liu K. Tang Y. Han X. (2022). Gallic acid inhibits mesaconitine-activated TRPV1-channel-induced cardiotoxicity. Evid. Based Complement. Altern. Med. 2022, 5731372. 10.1155/2022/5731372 PMC9020955 35463061

[B12] Henzi V. Reichling D. B. Helm S. W. MacDermott A. B. (1992). L-proline activates glutamate and glycine receptors in cultured rat dorsal horn neurons. Mol. Pharmacol. 41, 793–801. 10.1016/s0026-895x(25)09065-0 1349155

[B13] Honarvar N. Sheikhha M. H. Farashahi Yazd E. Pashaiefar H. Mohtaram S. Sazegari A. (2016). KDR gene polymorphisms and idiopathic recurrent spontaneous abortion. J. Matern. Fetal Neonatal Med. 29, 3737–3740. 10.3109/14767058.2016.1142966 26866667

[B14] Huang X. Yin T. Song M. Pan J. (2024). Association of estrogen receptor and progesterone receptor genetic polymorphisms with recurrent pregnancy loss: a systematic review and meta-analysis. Eur. J. Obstet. Gynecol. Reprod. Biol. 296, 65–75. 10.1016/j.ejogrb.2024.01.008 38402782

[B15] Ji L. Jiao Z. Zhang L. Shi J. Wan Q. Qian C. (2024). Role of increased IGFBP2 in trophoblast cell proliferation and recurrent spontaneous abortion development: a pilot study. Physiol. Rep. 12, e15939. 10.14814/phy2.15939 38316422 PMC10843903

[B16] Jiang L. Bu D. Zhao W. Zhang L. (2011). Clinical study on the treatment of habitual miscarriage with self-preservation drink prepared by Prof. Zhang Liangying. Yunnan J. Traditional Chin. Med. Materia Medica, 1–3. 10.16254/j.cnki.53-1120/r.2011.11.001

[B17] Karachrysafi S. Georgiou P. Kavvadas D. Papafotiou F. Isaakidou S. Grammatikakis I. E. (2023). Immunohistochemical study of MMP-2, MMP-9, EGFR and IL-8 in decidual and trophoblastic specimens of recurrent pregnancy loss cases. J. Matern. Fetal Neonatal Med. 36, 2218523. 10.1080/14767058.2023.2218523 37258409

[B18] Khadzhieva M. B. Lutcenko N. N. Volodin I. V. Morozova K. V. Salnikova L. E. (2014). Association of oxidative stress-related genes with idiopathic recurrent miscarriage. Free Radic. Res. 48, 534–541. 10.3109/10715762.2014.891735 24499375

[B19] Khosravi F. Zarei S. Ahmadvand N. Akbarzadeh-Pasha Z. Savadi E. Zarnani A.-H. (2014). Association between plasminogen activator inhibitor 1 gene mutation and different subgroups of recurrent miscarriage and implantation failure. J. Assist. Reprod. Genet. 31, 121–124. 10.1007/s10815-013-0125-8 24189965 PMC3909138

[B20] Kolben T. M. Rogatsch E. Hester A. Kuhn C. Schmoeckel E. Czogalla B. (2019). Involvement of ILR4α and TLR4 in miscarriages. J. Reprod. Immunol. 131, 36–43. 10.1016/j.jri.2018.12.001 30639993

[B21] Li A. Zhao M. Lin Z. Yang Z. Gong P. Wang C. (2025). Reduced SMEK1 regulates trophoblast migration and invasion in fetal growth restriction. Placenta 161, 65–75. 10.1016/j.placenta.2025.02.005 39929058

[B22] Li C. Zhang X. Kang X. Chen C. Guo F. Wang Q. (2020). Upregulated TRAIL and reduced DcR2 mediate apoptosis of decidual PMN-MDSC in unexplained recurrent pregnancy loss. Front. Immunol. 11, 1345. 10.3389/fimmu.2020.01345 32695113 PMC7338483

[B23] Li L. Liu J. Qin S. Li R. (2018). The association of polymorphisms in promoter region of MMP2 and MMP9 with recurrent spontaneous abortion risk in Chinese population. Med. Baltim. 97, e12561. 10.1097/MD.0000000000012561 PMC6200545 30290617

[B24] Li M. Haixia Y. Kang M. An P. Wu X. Dang H. (2021). The arachidonic acid metabolism mechanism based on UPLC-MS/MS metabolomics in recurrent spontaneous abortion rats. Front. Endocrinol. (Lausanne) 12, 652807. 10.3389/fendo.2021.652807 33868179 PMC8050334

[B25] Li X. Liu Z. Liao J. Chen Q. Lu X. Fan X. (2023). Network pharmacology approaches for research of Traditional Chinese Medicines. Chin. J. Nat. Med. 21, 323–332. 10.1016/S1875-5364(23)60429-7 37245871

[B26] Lu J. Zhang Y. Wang Y.-Z. Li Y.-Y. Wang R. Zhong Y.-J. (2022). Caffeic acid dimethyl ether alleviates alcohol-induced hepatic steatosis via microRNA-378b-mediated CaMKK2-AMPK pathway. Bioengineered 13, 11122–11136. 10.1080/21655979.2022.2060586 35481488 PMC9208468

[B27] McNamee K. Dawood F. Farquharson R. (2012). Recurrent miscarriage and thrombophilia: an update. Curr. Opin. Obstet. Gynecol. 24, 229–234. 10.1097/GCO.0b013e32835585dc 22729089

[B28] Meng D. Chen X.-J. Bian Y.-Y. Li P. Yang D. Zhang J.-N. (2005). Effect of astragalosides on intracellular calcium overload in cultured cardiac myocytes of neonatal rats. Am. J. Chin. Med. 33, 11–20. 10.1142/S0192415X05002618 15844829

[B29] Miljanović O. Ilić V. Teofilov S. Cikota-Aleksić B. Magić Z. (2023). Polymorphisms of ACE and thrombophilic genes: risk for recurrent pregnancy loss. J. Clin. Pathol. 76, 832–838. 10.1136/jcp-2021-208057 37977651

[B30] Nawrocka D. Krzyscik M. A. Opaliński Ł. Zakrzewska M. Otlewski J. (2020). Stable fibroblast growth factor 2 dimers with high pro-survival and mitogenic potential. Int. J. Mol. Sci. 21, 4108. 10.3390/ijms21114108 32526859 PMC7312490

[B31] Niu M. Zhang S. Zhang B. Yang K. Li S. (2021). Interpretation of network pharmacology evaluation method guidance. Chin. Traditional Herb. Drugs, 4119–4129. 10.7501/j.issn.0253-2670.2021.14.001

[B32] Osuga Y. Harada K. Tsuboi T. (2022). Identification of a regulatory pathway of L-phenylalanine-induced GLP-1 secretion in the enteroendocrine L cells. Biochem. Biophys. Res. Commun. 588, 118–124. 10.1016/j.bbrc.2021.12.043 34953208

[B33] Pan H. Dai H. (2021). A comparative study of the clinical effects of fetal preservation drink and low-dose aspirin in antiphospholipid antibody-associated recurrent miscarriage. Chin. J. Clin. Obstetrics Gynecol., 313–314. 10.13390/j.issn.1672-1861.2021.03.036

[B34] Peng L. Chelariu-Raicu A. Ye Y. Ma Z. Yang H. Ishikawa-Ankerhold H. (2021). Prostaglandin E2 receptor 4 (EP4) affects trophoblast functions via activating the cAMP-PKA-pCREB signaling pathway at the maternal-fetal interface in unexplained recurrent miscarriage. Int. J. Mol. Sci. 22, 9134. 10.3390/ijms22179134 34502044 PMC8430623

[B35] Qu H. Khalil R. A. (2020). Vascular mechanisms and molecular targets in hypertensive pregnancy and preeclampsia. Am. J. Physiol. Heart Circ. Physiol. 319, H661-H681–H681. 10.1152/ajpheart.00202.2020 32762557 PMC7509272

[B36] Quenby S. Gallos I. D. Dhillon-Smith R. K. Podesek M. Stephenson M. D. Fisher J. (2021). Miscarriage matters: the epidemiological, physical, psychological, and economic costs of early pregnancy loss. Lancet 397, 1658–1667. 10.1016/S0140-6736(21)00682-6 33915094

[B37] Shen Y. (2017). Analysis on the clinical effect of the treatment of early preeclampsia with the addition and subtraction of Foetus Preserving Drink. Electron. J. Clin. Med. Literature, 17956–17957. 10.16281/j.cnki.jocml.2017.91.074

[B38] Sun Y. Du H. Li R. Hao G. Feng X. Yu C. (2024). Guideline for diagnosis and treatment of recurrent spontaneous abortion with integrated traditional Chinese and western medicine. CHINA J. Chin. METERIA MEDICA, 2544–2556. 10.19540/j.cnki.cjcmm.20240130.501 38812150

[B39] Tian F. Zhang Y. Chen L. Wu J. Cao H. Wu M. (2025). Comprehensive quality evaluation and botanical differentiation of typhae pollen using UHPLC-DAD/Q-TOF-MS and multivariate chemometric analysis. Phytochem. Anal. 10.1002/pca.3519 39980040

[B40] van Wely M. (2023). Series of overviews on miscarriage and recurrent miscarriage. Fertil. Steril. 120, 932–933. 10.1016/j.fertnstert.2023.09.006 37722471

[B41] Walker V. (2025). The molecular biology of placental transport of calcium to the human foetus. Int. J. Mol. Sci. 26, 383. 10.3390/ijms26010383 39796238 PMC11720126

[B42] Yao R.-Q. Ren C. Xia Z.-F. Yao Y.-M. (2021). Organelle-specific autophagy in inflammatory diseases: a potential therapeutic target underlying the quality control of multiple organelles. Autophagy 17, 385–401. 10.1080/15548627.2020.1725377 32048886 PMC8007140

[B43] Yousefian M. Angaji A. Siasi E. Ali Rahmani S. Abbasalizadeh Khiaban S. (2022). Role of CYP1A1, CYP2D6, and NOS3 gene polymorphisms in idiopathic recurrent pregnancy loss in the Iranian Azeri population: a case-control study. Int. J. Reprod. Biomed. 20, 671–682. 10.18502/ijrm.v20i8.11756 36313260 PMC9596927

[B44] Zhang Y. Lai Y. Luo S. Gao J. (2022). Effects of jianwei shoutai pills on treg/Th17 cells balancing in abortion model rats. J. Traditional Chin. Med., 1271–1275. 10.13288/j.11-2166/r.2022.13.014

[B45] Zhao J. Chen P. Xu G. Sun J. Ruan Y. Xue M. (2023). Bushen Huoxue Fang improves recurrent miscarriage in mice by down-regulating the JAK2/STAT3 pathway. Nan Fang. Yi Ke Da Xue Xue Bao 43 (02.15), 265–270. 10.12122/j.issn.1673-4254.2023.02.15 36946047 PMC10034533

