# Supplementary figures and images for: Role of Ca2+/calmodulin and PI3K/AKT signaling pathways and active ingredients of BaoTaiYin in treatment of recurrent miscarriage
Source: Front Mol Biosci. 2025 Mar 25;12:1573294. doi: 10.3389/fmolb.2025.1573294 (PMC11975862; doi:10.3389/fmolb.2025.1573294)

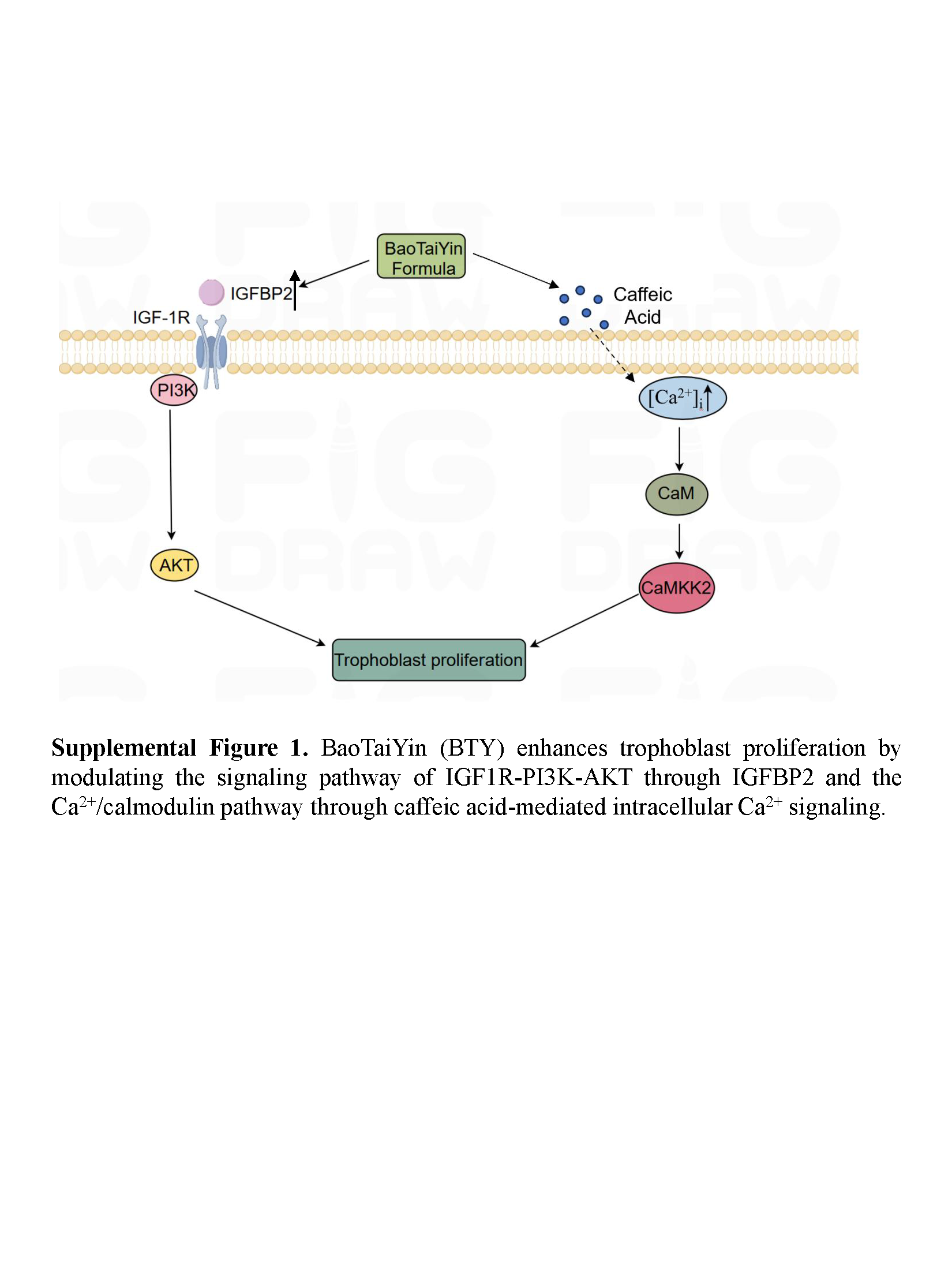

Supplement: Supplementary file 1 [file Image1.tif]
